# Collagen microsphere serving as a cell carrier supports oligodendrocyte progenitor cell growth and differentiation for neurite myelination *in vitro*

**DOI:** 10.1186/scrt320

**Published:** 2013-09-09

**Authors:** Li Yao, Francis Phan, Yongchao Li

**Affiliations:** 1 Department of Biological Sciences, Wichita State University, Wichita, KS, USA

**Keywords:** Microsphere, Oligodendrocyte, Cell delivery, Co-culture, Neural regeneration, Dorsal root ganglion

## Abstract

**Introduction:**

Microspheres fabricated from natural materials serve as a promising biodegradable and biocompatible carrier in a small volume for efficient cell delivery to the lesion of the injured neural tissue to generate biological functions. As the major component of extracellular matrix and due to its natural abundance within the body, collagen may be fabricated into microspheres and improve the ability of pre-seeded cells on the microspheres to encounter the hostile micro-environment in the lesion.

**Methods:**

In this study, collagen microspheres were fabricated using the water-in-oil emulsion technique and cross-linked with 1-ethyl-3-(3-dimethylaminopropryl) carbodiimide. Oligodendrocyte progenitor cells isolated from postnatal day P1 to 2 rats were cultured and differentiated on the microspheres. The microspheres carrying the oligodendrocyte progenitor cells were co-cultured with dorsal root ganglions from 15-day-old rat embryos. The myelination formation was studied for the co-culture of oligodendrocyte progenitor cells and dorsal root ganglions.

**Results:**

We showed that the viability of oligodendrocyte progenitor cells, B104 cells and PC12 cells grown on microspheres was not significantly different with those in cell culture plates. Oligodendrocyte progenitor cells differentiated into oligodendrocytes on collagen microspheres. The oligodendrocytes grown on microspheres extended processes that wrapped the axons of dorsal root ganglion neurons and the formation of myelin sheath was observed in the co-culture.

**Conclusions:**

This study demonstrates the feasibility of collagen microspheres in further applications for the delivery of neural progenitor cells for neural regeneration.

## Introduction

Oligodendrocytes (OLs) undergo both necrosis and apoptosis shortly after spinal cord injury (SCI). Loss of OLs causes demyelination and thereby impairs axon function and survival [1-3]. In response to demyelination, the oligodendrocyte progenitor cells (OPCs), which produce the proteoglycan NG2 and express the platelet-derived growth factor-α receptor on the cell membrane, are recruited from gray and white matter and migrate to the demyelinated site [4]. Direct evidence showed that OPCs will replace mature OLs after SCI and differentiate into OLs that eventually myelinate the axon [5]. Axon remyelination after SCI typically begins around two weeks post-injury and the endogenous oligogenic response is insufficient against OL loss and demyelination post-SCI. Transplantation of OPCs is potentially an efficient approach to remyelinate regenerating axons and to recover the spinal cord function after injury [6,7]. However, the microenvironment that the transplanted cells will encounter is the major concern in the process. Previous study showed that very few neural stem cells (NSCs) survive if directly transplanted to the site of damage [8]. Biomaterial scaffold delivery of the stem cells for the treatment of injured spinal cord improved the survival of the transplanted cells [9,10].

Micro- and nano-scale carriers fabricated from various natural and synthetic polymers have shown significant potential in biomedical and pharmaceutical applications. To encapsulate biomolecules in the particles to be delivered to the target tissues, the particles are able to generate a significant biological effect through a small volume [11-14]. Cell delivery and transplantation to the targeting tissue is an alternative application of the particles for the delivery of biomolecules. The investigation of microspheres for stem cell growth was reported in previous studies, which demonstrated a great potential of microspheres in stem cell delivery for tissue regeneration [15-17]. However, the feasibility of microspheres for neural progenitor cell growth for the repair of central nervous system has not been studied. Microspheres may provide the neural progenitor cells pre-seeded on the microspheres an optimal condition for the cells to adapt to the hostile environment in the lesion. The investigation of the growth, differentiation and ability for myelinating neurites of oligodendrocyte progenitor cells on microspheres will provide valuable information for its application in *in vivo* neural regeneration studies.

Collagen is the major component of extra-cellular matrix. Due to its natural abundance within the animal body, and its biodegradability and biocompatibility, collagen has been fabricated into microspheres and investigated for gene, growth factors and stem cell delivery [17-19]. Microspheres fabricated from collagen may work as efficient carriers for oligodendrocyte progenitor cell proliferation and differentiation, and can be potentially used to deliver OPCs to myelinate regenerating spinal axons of the injured spinal cord. In this study, we will investigate the growth and differentiation of OPCs on collagen microspheres and study myelination of the axons of dorsal root ganglion (DRG) neurons by OPCs carried by collagen microspheres *in vitro*. This study will provide us with important information for the further study of repairing the injured spinal cord using collagen microspheres delivering OPCs in the animal model.

## Methods

### Collagen microsphere fabrication

The collagen microspheres were fabricated as reported [19]. In brief, Type I collagen (extracted from bovine Achilles tendon) solution (5 mg/ml) emulsified in liquid paraffin oil (Sigma-Aldrich, St. Louis, MO, USA) containing surfactant (Span 20, Sigma-Aldrich) (0.3% (v/v)) and stirred using a ceramic-top stirring plate (Fisher Scientific, Pittsburg, PA, USA) for five minutes. 1-ethyl-3-(3-dimethylaminopropryl) carbodiimide (EDC) (Sigma-Aldrich) (50% in water (v/v)) was added to the emulsified mixture, and was stirred for 1 h to form collagen microspheres. Ethanol (50% (v/v) in water) was then added into the mixture and stirred for five minutes to separate the collagen microspheres from the oil phase. The mixture was then centrifuged at 3,500 rpm for five minutes. This step was repeated three times to remove the supernatant. The collagen microspheres were washed with sterile phosphate-buffered saline (PBS) and centrifuged again (3,500 rpm five minutes) to remove the supernatant. Sterile PBS was added to the centrifuge tube to re-suspend the microspheres and the tube with the microsphere suspension was stored at 4°C before the cells were seeded. In the studies of cell viability assay and the co-culture of microspheres delivered OPCs and DRGs, 20% of microspheres generated by 1 ml collagen solution were placed in each well of a 48-well plate. The microspheres fully covered the bottom of the well and cells were seeded on top of the microspheres. To study the OPCs’ growth and differentiation on collagen microspheres with immunocytochemistry, 5% of the microspheres generated by 1 ml collagen solution were placed in each well of a 48-well plate. The immunolabeled cells on microspheres and on a cell culture plate can be easily visualized with the low density of the microspheres in the cell culture wells.

### OPCs isolation and culture

Procedures used in animal experiments were approved by the Institutional Animal Care and Use Committee (IACUC) and completed at the Wichita State University, Wichita, KS, USA. OPC cultures were established as reported previously [20]. In brief, postnatal day P1 to 2 rats were sacrificed and cerebral cortexes were isolated from the brain. The cortex tissues were each triturated gently and sequentially twice through needles with a 1-cc syringe. The tissue suspension was passed through a 70 mm nylon cell strainer (BD Falcon™, Durham, NC, USA) which was placed on a 50 ml conical tube and the flow-through was collected. The isolated cells were cultured for about seven days. The OPCs were then isolated from the mixed cell culture layer by mechanically shaking the cell culture flasks. The collected OPCs were cultured in cell culture dishes coated with Poly-D,L-ornithine (5 mg/ml in PBS, Sigma-Aldrich). The OPCs were fed with OPC growth medium containing DMEM (Life Technologies™, Grand Island, NY, USA), Sato media (DMEM, 100 μg/ml transferrin, 100 μg/ml BSA, 0.2 μM progesterone, 16 μg/ml putrescine, 40 ng/ml sodium selenite (Sigma-Aldrich)), 1% penicillin-streptomycin, 2 mM L-glutamine (Life Technologies™), 5 μg/ml insulin, 10 nM D-biotin, 1 mM sodium pyruvate, 5 μg/ml N-acetyl cysteine (Sigma-Aldrich), Trace Elements B (1x, Mediatech, Inc., Manassas, VA, USA) and 10 ng/ml PDGF and 10 ng/ml bFGF (Peprotech, Rocky Hill, NJ, USA). Then the OPCs were passaged or grown on collagen microspheres.

### Cell viability assays

The viability and proliferation of OPCs, B104 cells and PC12 cells, which were seeded on collagen microspheres, were studied by monitoring their metabolic activity using alamarBlue assay (Pierce Biotechnology, Rockford, IL, USA). To perform the alamarBlue assay, the sterile PBS containing collagen microspheres was centrifuged and the microspheres were resuspended in the cell culture medium. The collagen microspheres were placed into the 48-well plates and the bottom of the cell culture wells were fully covered by the microspheres. After the microspheres were settled down at the bottom of the cell culture wells, the cells (15,000 cells/well) were added into the cell culture medium and the cells grew on the microspheres. In the control study, the cells (15,000 cells/well) were directly seeded in 48-well plates coated with poly-D,L-ornithine (5 mg/ml in PBS). After culturing for four days, the medium was gently removed from the wells and the cells were then incubated with culture medium containing 10% (v/v) alamarBlue reagent for two hours. Then the cell culture medium containing alamarBlue reagent was transferred to a 96-well plate (100 μl/well) and absorbance was measured at a wavelength of 570 nm and 600 nm in a microplate reader (Synergy Mx Monochromator-Based Multi-Mode Microplate Reader, Winooski, VT, USA). The cell viability of the 3T3 cells and PC12 cells growing on the collagen microspheres was analyzed by fluorescence-based LIVE/DEAD® assays (Life Technologies™). The Calcein and EthD-1 labeled cells were counted and quantified to show the rate of live cells on the collagen microspheres.

### DRG isolation and culture

DRGs from 15-day-old Sprague Dawley rat embryos were isolated and cultured in the collagen-coated 24-well-plate wells [21]. The whole DRGs were cultured in Neurobasal media supplemented with B27, 1% penicillin/streptomycine, 2 mM L-glutamine (Life Technologies™), 20 ng/ml 2.5S nerve growth factor (NGF, Alomone Labs, Jerusalem, Israel), 8.625 μg/ml uridine and 3.75 μg/ml of 5-fluoro-2- deoxyuridine (Sigma-Aldrich). The DRGs were cultured for 10 days before they were co-cultured with OPCs.

### OPCs and DRG co-culture

OPCs (15,000 cells/well) were seeded on the collagen microspheres and cultured with OPCs growth medium for two days. The collagen microspheres with pre-seeded OPCs were then transferred into the wells with the growth of DRGs [22]. In the control group, OPCs (15,000 cells/well) were added into the DRG culture wells. The co-culture of the DRG and OPCs were maintained for eight days in the DMEM with Sato media, 1% penicillin-streptomycin, 2 mM L-glutamine, 5 μg/ml insulin, 10 nM D-biotin, 1 mM sodium pyruvate, 5 μg/ml N-acetyl cysteine, Trace Elements B and Triiodothyronine (Sigma-Aldrich). Negative control was performed to treat the cultured DRG with the co-culture medium without adding OPCs in the cell culture.

### Immunocytochemistry

OPCs and DRGs were fixed with 4% paraformaldehyde in PBS solution. The phenotype of the cultured OPCs was determined with anti-A2B5 antibody (a gift from Dr Qing Lu, University of Texas Southwestern Medical Center). The differentiated OPCs were labeled with anti-O4 antibody (a gift from Dr Qing Lu, University of Texas Southwestern Medical Center) and anti-Myelin Basic Protein (MBP) antibody (Millipore, Billerica, MA, USA). Dual labeling of DRG neuron axons and oligodendrocyte were performed to study the myelination of the DRG axons using anti-IIIβ-tubulin antibody (Millipore) and anti-MEB antibody, respectively. The images were taken using Zeiss Axio Observer microscope (Carl Zeiss Microscopy, LLC, Thornwood, NY, USA).

### Statistics

Statistical analysis was conducted using a two-tailed Student’s *t*-test. A *P*-value of 0.05 was considered to be statistically significant. Data are expressed as means ± standard deviation.

## Results

### Collagen microspheres supported the proliferation of the cells grown on the surface of the microspheres

**Figure 1:**
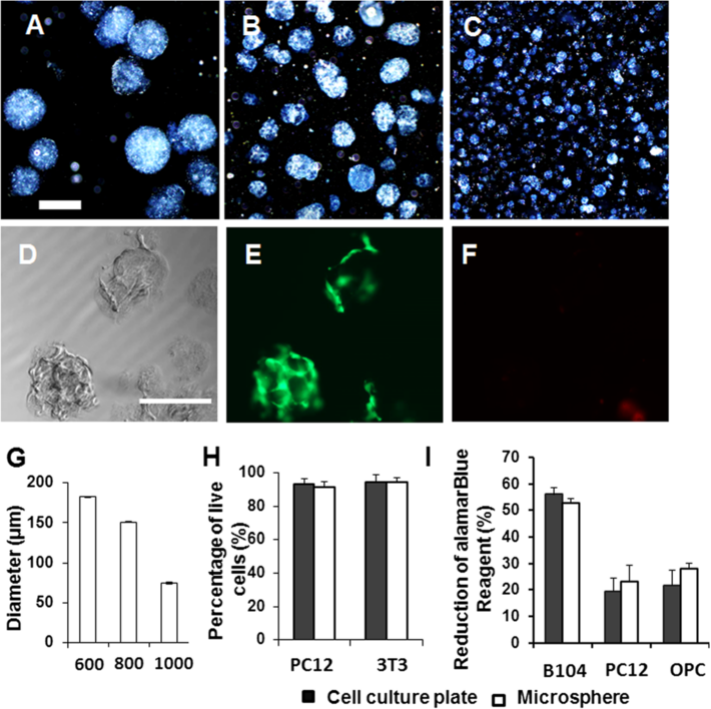
**Cell viability of the cells grown on the collagen microspheres. (A-C)** Fabricated collagen microspheres. The diameter of collagen microspheres increased when the stirring speed for the mixture of collagen solution, paraffin oil and surfactant was increased. The stirring speeds for the fabrication of collagen microspheres are 600 rpm **(A)**, 800 rpm **(B)** and 1,000 rpm **(C)**. Scale bar: 200 μm. **(D-F)** 3T3 cells that grew on collagen microspheres were labeled with Calcein **(D)** and Ethidium **(E)** in the LIVE/DEAD® cell viability assay. **(G)** Quantification of microsphere diameter fabricated at different stirring speed. **(H)** Quantification of live cells of PC12 cells and 3T3 cells grown in cell culture plate or on collagen microspheres. Scale bar: 200 μm **(I)** alamarBlue cell viability assay of B104 cells, PC12 cells and oligodendrocyte progenitor cells (OPCs) growing in cell culture plate or on collagen microspheres.

Collagen microspheres were fabricated using the water-in-oil emulsion technique by adding collagen and cross-linking reagent to a solution containing paraffin oil and surfactant, and stirring the mixture using a stirring plate (Figure 1A-C). The diameter of the collagen microspheres decreased from 182.3 ± 8.8 μm to 74.4 ± 3.1 μm (n = 3, mean ± SD, *P* <0.01), when the stirring speed increased from 600 rpm to 1,000 rpm (Figure 1G).

The LIVE/DEAD® assay and alamarBlue assay were performed to analyze the cell viability of the cells grown on the collagen microspheres. The LIVE/DEAD® assay showed that most 3T3 cells that grew on the surface of collagen microspheres were live cells (Figure 1D-F). Further quantification showed that after being cultured for three days, the rate of live cells of 3T3 cells and PC12 cells was 91.5 ± 4.3% and 94.3 ± 2.9% (n = 3, mean ± SD), respectively, which is not significantly different from that of the cells that grew in cell culture dishes (Figure 1H). The alamarBlue assay showed that the reduction of alamarBlue reagent for the OPCs, B104 cells and PC12 cells grown on collagen microspheres was 27.9 ± 2.2%, 52.9 ± 1.6%, and 23.1 ± 6.2%, (n = 4, mean ± SD), respectively, which was not significantly different from that on cell culture plates (OPCs, 21.7 ± 5.5%; B104 cells, 56.1 ± 2.4%; PC12 cells, 19.5 ± 5.1%) (n = 4, mean ± SD) (Figure 1I).

### Collagen microspheres support the differentiation of OPCs grown on the microspheres

**Figure 2:**
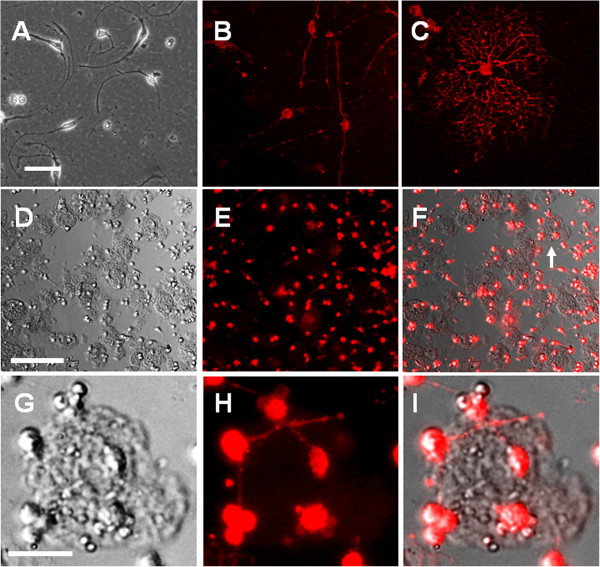
**Growth of OPCs on collagen microspheres. (A-C)** Oligodendrocyte progenitor cells (OPCs) grown in cell culture plate were labeled with anti-A2B5 antibody. Scale bar: 100 μm. **(D-F)** Bright field and fluorescent images showed the OPCs grown on collagen microspheres. The OPCs are labeled with anti-A2B5 antibody. Scale bar: 200 μm. **(G-I)** A typical microsphere with the growth of OPCs highlights the morphology of OPCs grown on microspheres. Scale bar: 50 μm.

The OPCs grown in the cell culture plate developed multiple processes and were labeled with anti-A2B5 antibody. The phenotype and morphology of the OPCs growing on collagen microspheres were also studied. OPCs were seeded on the collagen microspheres and cultured with OPC medium for three days. Then the growth of OPCs on the collagen microspheres was observed. Immuno-labeling with anti-A2B5 antibody showed that the OPCs expressed A2B5 antigen and the OPCs on collagen microspheres developed short processes (Figure 2).

**Figure 3:**
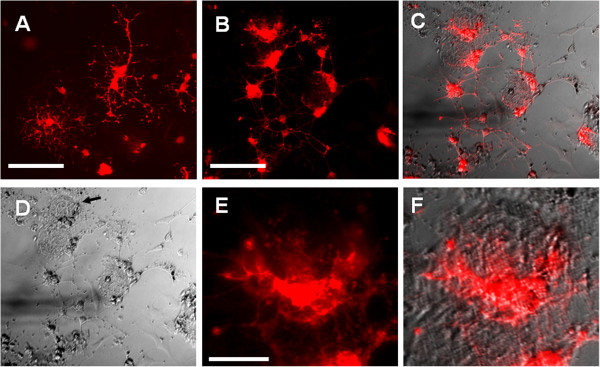
**The differentiation of OPCs on collagen microspheres. (A)** Differentiated oligodendrocyte progenitor cells (OPCs) in cell culture plates are labeled with anti-MBP antibody. Scale bar: 200 μm. **(B-D)** Differentiated OPCs grown on collagen microspheres are labeled with anti-myelin basic protein (MBP) antibody. Scale bar: 200 μm. **(E)** and **(F)** The morphology of differentiated OPCs on a collagen microsphere. Scale bar: 50 μm.

After being cultured for eight days in differentiation medium, the OPCs differentiated into oligodendrocytes on collagen microspheres and in cell culture dishes (Figure 3). The differentiated cells in cell culture plates expressed MBP protein and were labeled with anti-MBP antibody. The differentiated OPCs grown on collagen microspheres developed multiple processes and were also labeled with anti-MBP antibody. The multiple processes of oligodendrocytes grown on collagen microspheres surrounded the collagen microspheres. The oligodendrocyte growing on the collagen microspheres also extended processes to the culture dishes and established connections with other oligodendrocytes (Figure 3E, F).

### OPCs seeded on collagen microspheres differentiated and formed myelin sheaths for neurites of the cultured DRG neurons

**Figure 4:**
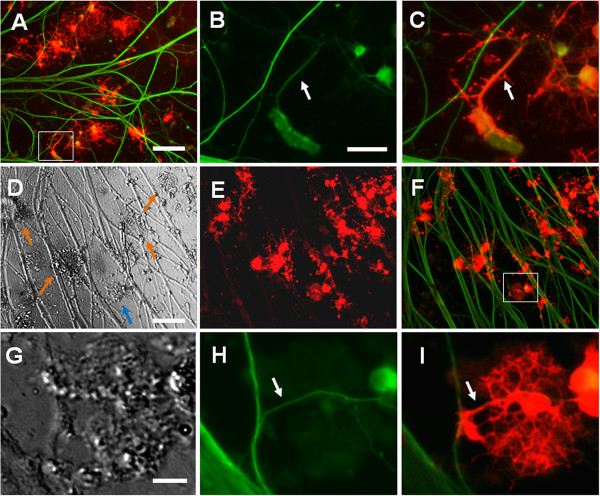
**Co-culture of DRGs and OPCs carried by collagen microspheres. (A)** Co-culture of dorsal root ganglions (DRGs) with oligodendrocyte progenitor cells (OPCs) in cell culture plate. Scale bar: 200 μm. **(B)** and **(C)** Magnified images shows the oligodendrocytes (OLs) wrap DRG neurites. Scale bar: 50 μm. **(D-F)** Co-culture of DRGs and OPCs carried by collagen microspheres. Scale bar: 200 μm. **(D)** Bright field image shows that collagen microspheres attach to the cell culture plate. The arrows point at the collagen microspheres. **(E)** and **(F)** OPCs on collagen microspheres differentiated into OLs. The processes of OLs wrapped the neurites of DRGs. **(G-I)** Magnified images for the microsphere indicated by blue arrow in **(D)** show the irregular shape of the collagen microsphere and the neurites of DRGs were wrapped by OLs. Scale bar: 100 μm.

After the co-culture of OPCs and DRGs was maintained for eight days, OPCs differentiated and developed multiple processes, which associated with the neurites of DRG neurons. In this study, myelin sheath formation around the neurites of DRG neurons was observed (Figure 4B, C). Collagen microspheres pre-seeded with OPCs were transferred to the cell culture dishes with the growth of DRGs and the culture was maintained for eight days. Some collagen microspheres attached to the neurites of the DRG neurons. Oligodendrocytes developed multiple processes on the collagen microspheres (Figure 4D). The authors observed that multiple processes from the oligodendrocytes grown on microspheres wrapped the DRGs’ neurites (Figure 4E) and the myelin sheath formation around the neurites of the DRG neurons (Figure 4H). In the negative control, DRGs were cultured in co-culture medium without adding OPCs in the cell culture. After the DRGs were cultured for eight days, we did not see any MBP positive cells in the cell culture.

## Discussion

In this study, collagen microspheres were fabricated using the water-in-oil emulsion technique and cross-linked with EDC. We showed that the viability of oligodendrocyte progenitor cells, B104 cells and PC12 cells grown on collagen microspheres was not significantly different from that in the cell culture plates. We also showed that OPCs differentiated into oligodendrocytes on collagen microspheres. The OPCs were then grown on collagen microspheres and co-cultured with DRGs. We demonstrated that the oligodendrocytes grown on microspheres extended processes that wrapped the axons of DRGs and the myelin sheath formation for the neurites of DRG neurons was observed.

Microspheres fabricated from biocompatible materials provide a supportive environment for cell growth, while the cells growing on the microspheres can also modify the structure of the microspheres. Previous work showed that the ability of porous chitosan microspheres can support adipose-derived stem cell growth [16]. When the spheres were incubated with adipose-derived stem cells (ADSCs), the spheres proliferated on the surface of the spheres, as well as infiltrated the pores to grow within the spheres as well. The cells growing on spheres maintained their metabolic activity and viability. Because of its biological and structural properties, type I collagen has been processed as an engineered scaffold for neural regeneration [23-25]. EDC cross-linked neural conduits were fabricated in previous studies and their biocompatibility was shown by growing dorsal root ganglion in the cross-linked collagen conduits [21]. It was also demonstrated that the EDC cross-linked collagen conduits supported peripheral nerve regeneration after the implantation of neural conduits in the transected rat sciatic nerve [26]. In this study, we showed the proliferation of OPCs, PC12 cells and B104 cells on collagen microspheres was not significantly different from that in cell culture plates. We observed that most collagen microspheres maintained round morphology after the OPCs were grown on the microspheres for three days. However, we found the morphology of the microspheres changed after the OPCs were cultured on the microspheres after eight days. Some round collagen microspheres changed to an irregular morphology. This suggests the OPCs can gradually re-organize the morphology of the collagen microspheres. In *in vivo* studies, it is likely that OPC-grown collagen microspheres will be transplanted into the wounded neural tissue after the OPCs are cultured on collagen microspheres for two to four days, as the OPCs can easily maintain their phenotype during the period *in vitro*. After two to four days’ culture, the microspheres should efficiently deliver the OPCs to the wounded neural tissue. The OPCs are expected to incorporate into the local tissue with the degradation of the collagen microspheres.

For the cell transplantation and therapy, stem cells delivered by the microspheres need to maintain their phenotype and their differentiation ability. We observed that OPCs showed the A2B5 marker in OPCs medium and differentiated into oligodendrocytes with differentiation medium when they were cultured on collagen microspheres. We found that the oligodendrocytes extended processes around the microspheres and established connection with other oligodendrocytes. This study suggests that collagen is a biocompatible material that can support OPC growth and differentiation. The cross-linking reagent EDC is not toxic to the OPCs. In this study, 15,000 cells were on microspheres of each well for viability assay and for co-culture with DRGs. The concern of significantly increasing the OPCs seeding density on microspheres is that OPCs cannot maintain the phenotype and differentiate into oligodendrocytes. The OPCs’ seeding density on microspheres for transplantation studies should be optimized in further *in vivo* studies. Different from the *in vitro* condition, after transplantation into the wounded neural tissue, OPCs may adhere to the surrounding tissue and grow and some of the OPCs may die off. The optimal OPCs’ density for transplantation should be higher compared with that for the current study.

Targeting the cell transplantation to the lesion and therapy, it is important to maintain the cell’s function in the process of cell encapsulation. In order to ensure this, the type of polymer used must fulfill appropriate mechanical durability, pore size, capsule consistency, degradation features and low immunogenicity [27]. Therefore, natural polymers are strongly favored because they are usually biodegradable, nontoxic and non-immunogenic. Previous study showed that collagen type I gel can support the growth of neural stem/precursor cells (NSPCs) and the NSPCs could differentiate into neurons, astrocytes and oligodendrocytes in the collagen gel [28]. In another study, OPCs were grown on coverslips coated with fibronectin, laminin or Matrigel and the results showed that these ECM components enhanced the biological properties of the OPCs compared with a non-ECM substrate [29]. Potentially, collagen and fibronectin can be fabricated as hydrogel or microspheres for OPCs delivery to treat the injured spinal cord. Different from the hydrogel approach, which is particularly suitable to fill neural defect, microspheres could be delivered with PBS to a lesion without significant neural defect because the size of the microspheres is relatively small. Additionally, as the collagen or fibronectin is mechanically elastic, collagen- or fibronectin-based microspheres will create little damage to the healthy tissue after implantation. Previous studies showed that the degradation property of the collagen scaffolds can be modified by changing cross-linking reagent concentration. The degradation rate of the collagen microspheres can be regulated by modifying the level of the cross-linking reagent. The degradation of the microspheres will be determined by *in vivo* study. The degradation rate can be optimized by studying the microsphere degradation rate and cell survival *in vivo*. The optimal degradation rate is the maximal OPCs cell survival with the removal of the microspheres from the host tissue.

## Conclusions

In this study we showed that collagen microspheres can support the growth and differentiation of OPCs that were seeded on the microspheres. Oligodendrocytes extended multiple processes that surrounded the collagen microspheres and wrapped the neurites of the DRGs. The formation of a myelin sheath was observed in the co-culture of DRGs and OPCs carried by collagen microspheres. This study demonstrates the potential of collagen microspheres in the applications of delivery and transplantation of neural stem cells for neural regeneration of in the central nervous system.

## Abbreviations

ADSCs: Adipose-derived stem cells; BSA: Bovine serum albumin; DMEM: Dulbecco’s modified Eagle’s medium; DRG: Dorsal root ganglion; EDC: 1-Ethyl-3-(3-dimethylaminopropryl) carbodiimide; IACUC: Institutional Animal Care and Use Committee; MBP: Myelin basic protein; NGF: Nerve growth factor; NSCs: Neural stem cells; NSPCs: Neural stem/precursor cells; OLs: Oligodendrocytes; OPCs: Oligodendrocyte progenitor cells; PBS: Phosphate buffered saline; SCI: Spinal cord injury.

## Competing interests

The authors declare that they have no competing interests.

## Authors’ contributions

LY conceived the idea and designed the experiments. LY also performed the primary cell culture, the myelination studies, data analysis and manuscript preparation. FP was responsible for the microsphere fabrication and cell viability studies. YCL performed immune-staining assays and data analysis. All authors read and approved the final manuscript.
